# Zugangsbarrieren zur Schwangerschaftsabbruchversorgung: Eine Analyse aus der Perspektive ungewollt Schwangerer – Erkenntnisse aus der Studie „Erfahrungen und Lebenslagen ungewollt Schwangerer. Angebote der Beratung und Versorgung (ELSA)“

**DOI:** 10.1007/s00103-024-03987-2

**Published:** 2024-12-04

**Authors:** Daphne Hahn, Rona Torenz, Ines Thonke, Sarah Eckardt, Maria Schneider, Anke Wyrobisch-Krüger, Ulrike Busch, Cornelia Helfferich, Tilmann Knittel, Maika Böhm, Petra Brzank, Christine Knaevelsrud, Silvia Krumm, Sarah Schumacher

**Affiliations:** 1 Fachbereich Gesundheitswissenschaften, Hochschule Fulda, Leipziger 123, 36037 Fulda, Deutschland; 2 Sozialwissenschaftliches Forschungsinstitut zu Geschlechterfragen – SOFFI F., Forschungs- und Innovationsverbund an der Evangelischen Hochschule Freiburg (FIVE e. V.), Freiburg, Deutschland; 3 Fachbereich Soziale Arbeit.Medien.Kultur, Hochschule Merseburg, Merseburg, Deutschland; 4 Fachbereich Wirtschafts- und Sozialwissenschaften, Hochschule Nordhausen, Nordhausen, Deutschland; 5 Fachbereich Erziehungswissenschaft und Psychologie, Freie Universität Berlin, Berlin, Deutschland; 6 Klinik für Psychiatrie und Psychotherapie II, Universitätsklinikum Ulm, Ulm, Deutschland

**Keywords:** Schwangerschaftsabbruch, Barrieren, Versorgungszugang, Erreichbarkeit, Verfügbarkeit, Stigmatisierung, Abortion, Barriers, Abortion care, Accessibility, Availability, Stigmatisation

## Abstract

**Einleitung:**

Barrieren beim Zugang zur Schwangerschaftsabbruchversorgung können die Inanspruchnahme von Versorgungsleistungen verzögern, was zu einem zeitlich verzögerten Schwangerschaftsabbruch und gesundheitlichen Risiken führen kann. Zu Barrieren gehören u. a. die räumliche Erreichbarkeit, Geheimhaltung, Wartezeiten, Stigmatisierung, schlechte oder schlecht zugängliche Informationen und Kosten für einen Abbruch. Im Beitrag werden Barrieren beim Zugang zur Schwangerschaftsabbruchversorgung in Deutschland untersucht. Eingeschlossen werden Barrieren in der Verfügbarkeit und Erreichbarkeit von Versorgungsangeboten, im Zugang zu Informationen, den Kosten für einen Abbruch sowie organisatorische Hindernisse.

**Methoden:**

Die Analysen basieren auf den Daten einer Online-Querschnittsbefragung von 594 Frauen mit Schwangerschaftsabbruch in Deutschland, die im Rahmen der ELSA-Studie 2021 und 2022 durchgeführt wurde.

**Ergebnisse:**

Die Ergebnisse zeigen, dass auch in Deutschland der Zugang zu einem Schwangerschaftsabbruch für viele Frauen mit unterschiedlichen Hürden verbunden ist. 80,1 % der Befragten gaben mindestens eine Barriere beim Zugang zum Schwangerschaftsabbruch an, 65,5 % mehr als 2 und 3 und mehr Barrieren wurden von 40,5 % der Befragten angegeben. Besonders die Geheimhaltung des Eingriffs und damit einhergehende Stigmatisierungsängste stellten für viele Teilnehmerinnen Hürden dar.

**Diskussion und Fazit:**

Die Studienergebnisse unterstreichen die Notwendigkeit, den Zugang zu sicheren Schwangerschaftsabbrüchen zu verbessern. Zum Abbau der Zugangsbarrieren gehören daher die Entkriminalisierung, eine flächendeckende Versorgungsstruktur, finanzielle Unterstützungsangebote, verbesserte Informationsangebote sowie der Abbau von Stigmatisierung und Diskriminierung.

## Abstract (en)

**Introduction:**

Barriers to accessing abortion care can delay access to services, which can lead to delayed abortion and health risks. Barriers include geographical accessibility, confidentiality, waiting times, stigmatization, poor or inaccessible information and the cost of abortion. This article examines barriers to accessing abortion care in Germany. This includes barriers in the availability and accessibility of care services, access to information, costs associated with abortion and organisational barriers.

**Methods:**

The analyses are based on data from an online cross-sectional survey of 594 women in Germany who had an unwanted pregnancy terminated, which was conducted as part of the ELSA Study in 2021 and 2022.

**Results:**

The findings indicate that access to abortion care in Germany is associated with various barriers for many women. Of the respondents, 80.1% reported at least one barrier to accessing abortion, 65.5% reported more than two barriers and 40.5% reported three or more barriers. In particular, the non-disclosure of the procedure and the associated fear of stigmatisation represented hurdles for many participants.

**Discussion and conclusion:**

The results of the study underline the need to improve access to safe abortions. Reducing access barriers therefore includes the decriminalisation of abortion, a comprehensive care structure, financial support services, improved information services and the reduction of stigmatisation and discrimination.

## Einleitung

Ungewollt Schwangere werden auf dem Weg zu einem Schwangerschaftsabbruch mit verschiedenen Barrieren konfrontiert [1]. Zu den Barrieren zählen Schwierigkeiten bei der Orientierung im Versorgungssystem, schlechte oder schwer zugängliche Informationen, begrenzte Auswahlmöglichkeiten von Abbrucheinrichtungen, staatliche Restriktionen wie Pflichtberatungen und Wartezeiten, Geheimhaltung, Stigmatisierung, Zeitdruck, ein großer organisatorischer Aufwand, fehlende Wahlmöglichkeit der Abbruchmethode, Kosten für einen Abbruch sowie der eingeschränkte Zugang zu Einrichtungen, die einen Abbruch vornehmen [2–16].

Von Zugangsbarrieren sind insbesondere Frauen aus vulnerablen Gruppen, zum Beispiel solche mit geringeren sozioökonomischen Ressourcen, Sprachbarrieren, gewalttätigen Partnern, Frauen mit Behinderungen, Minderjährige sowie Frauen ohne legalen Aufenthaltsstatus, betroffen, die aufgrund von Zugangsbarrieren häufiger auf nicht legale Angebote zurückgreifen [17, 18]. Barrieren beim Zugang können die Inanspruchnahme von Versorgungsleistungen verzögern, führen zu schlechterem Wohlbefinden und gehen mit größeren psychischen und physischen Belastungen sowie größeren gesundheitlichen Risiken einher [1]. Verzögerungen können außerdem den Zugang zu einem frühen Schwangerschaftsabbruch und zur zeitlich stärker befristeten medikamentösen Methode verhindern [19]. Je früher der Abbruch nach der Feststellung einer Schwangerschaft erfolgt, desto mehr Versorgungsoptionen stehen den Schwangeren zur Verfügung und desto handlungsfähiger fühlen sie sich [5, 7, 20]. Haben ungewollt Schwangere wenige Versorgungsoptionen und erleben viele Barrieren, erhöhen sich der organisatorische Aufwand und die Kosten [11–13]. Auch sind Wohlbefinden und Zufriedenheit geringer und psychische Belastungen aufgrund von Zeitdruck größer [11–13]. Frauen mit einem Schwangerschaftsabbruch sind umso zufriedener, je größer der wahrgenommene Gestaltungsspielraum und die Wahlmöglichkeiten hinsichtlich der Abbrucheinrichtung, der Abbruchmethode und des organisatorischen Aufwandes sind [7, 20, 21]. Umgekehrt ist die Belastung größer, wenn die Abbrucheinrichtung schwerer erreichbar und der organisatorische sowie finanzielle Aufwand für die Frauen höher ist [2]. Ungewollte Schwangerschaften werden eher geheim gehalten, wenn Frauen sie nicht austragen wollen [22]. Die Geheimhaltung wiederum geht oft mit Ängsten und erwarteter Stigmatisierung einher [22]. Je größer die Angst vor negativer Bewertung, Beeinflussung oder auch negativen Konsequenzen durch das Umfeld ist, desto größer sind der Wunsch und die Bemühungen, die Schwangerschaft und den Schwangerschaftsabbruch geheim zu halten, was wiederum den Zugang zu Versorgung erschwert [5, 6].

Ziel des hier vorgestellten Teils der ELSA-Studie ist, den Zugang zu medizinischer Versorgung zum Schwangerschaftsabbruch aus der Perspektive von Frauen zu untersuchen und darzustellen, welche Barrieren durch ungewollt Schwangere wahrgenommen werden.

### Konzeptioneller Rahmen und theoretische Einordnung

Die Erfahrungen, die Frauen von der Feststellung einer Schwangerschaft bis zum Schwangerschaftsabbruch machen, weisen im Vergleich mit anderen medizinischen oder fachärztlichen Dienstleistungen einige Besonderheiten auf. Die Übersichtsarbeit von Coast et al. [6] verdeutlicht das multidimensionale Geschehen anhand von Einflussfaktoren, die in 3 Kategorien eingeteilt werden: erstens abbruchspezifische Faktoren wie Scham- und Stigmagefühle, das soziale Umfeld oder eine (Nicht‑)Offenlegung. Unter die zweite Kategorie fallen individuelle Kontextfaktoren wie der sozioökonomische Status, das Alter oder die Partnerschaft [6]. Zur dritten Kategorie gehören die rechtliche Einbettung des Schwangerschaftsabbruches, das Gesundheitssystem und das Wissensumfeld [6]. Infolgedessen unterscheiden sich die Erfahrungen, die Frauen bei der Suche nach, dem Zugang zu und dem Umgang mit einem Schwangerschaftsabbruch machen [1, 23, 24].

Die Vielschichtigkeit der Versorgungsaspekte beim Schwangerschaftsabbruch wird auch in der aktuellen Leitlinie der Weltgesundheitsorganisation (WHO; [1]) deutlich. Die WHO formulierte evidenzbasierte Standards für eine umfassende Versorgung, zu denen ein niedrigschwelliger Zugang zum Schwangerschaftsabbruch sowie zu evidenzbasierten Informationen, medizinische Behandlungsstandards und auch die rechtliche Verankerung außerhalb des Strafrechts gehören.

Die vorliegende Untersuchung greift auf die theoretische Konzeptualisierung des Zugangs zur Gesundheitsversorgung zurück, die eine strukturierte Grundlage für die Analyse und Bewertung von Gesundheitsdienstleistungen bietet und ermöglicht, potenzielle Barrieren zu identifizieren. Zugang wird hier als Resultat der Interaktion zwischen den Merkmalen von Personen, sozialen und physischen Umgebungen sowie des Gesundheitssystems verstanden [25, 26]. Dazu gehört auch die Möglichkeit, eigene Bedürfnisse bzgl. der Gesundheitsversorgung zu erkennen, die notwendigen Versorgungsleistungen zu suchen, die dem individuellen Versorgungsbedarf entsprechen, sie zu finden sowie sie tatsächlich zu erhalten [27]. Die Basis der Systematisierung von Barrieren im Zugang zur Versorgung bei Schwangerschaftsabbruch der vorliegenden Untersuchung bilden die 5 Dimensionen des Zugangs von Penchansky und Thomas: „Availability“ (Verfügbarkeit), „Accessibility“ (Erreichbarkeit), „Accomodation“ (Passfähigkeit), „Affordability“ (Bezahlbarkeit) und „Acceptability“ (Annehmbarkeit; [25]).

## Methoden

Die Studie „Erfahrungen und Lebenslagen ungewollt Schwangerer. Angebote der Beratung und Versorgung – ELSA“ wird seit Oktober 2020 vom Bundesministerium für Gesundheit gefördert und endet im Oktober 2024. Ziel der Studie ist, Erkenntnisse über die sozialen und gesundheitlichen Belastungen und Ressourcen von Frauen zu gewinnen, die eine ungewollte Schwangerschaft austragen oder abbrechen. Darüber hinaus sollen in der Studie sowohl die aktuelle medizinische als auch die psychosoziale Angebotsstruktur untersucht werden. Auf der Grundlage der Ergebnisse sollen Schlussfolgerungen formuliert werden, die dazu dienen, die gesundheitliche und psychosoziale Versorgung ungewollt schwangerer Frauen verbessern zu können. An dem Verbundvorhaben sind 6 Forschungszentren beteiligt, die jeweils eigenständige Projektteile verantworten, die in wechselseitigen Bezügen zueinanderstehen. Die Hochschule Fulda verantwortet unter anderem den gesamten Untersuchungsteil zur medizinischen Versorgung bei Schwangerschaftsabbruch. In dem vorliegenden Beitrag werden Ergebnisse der Befragung von Frauen mit ungewollten abgebrochenen Schwangerschaften mit Fokus auf Barrieren in der medizinischen Versorgung dargestellt.

### Datenerhebung und -analyse

Zur Gewinnung von Daten zu ungewollten Schwangerschaften und zu Schwangerschaftsabbrüchen wurden mehrere standardisierte Befragungen von Frauen durchgeführt. Die Basiserhebung bildete eine für Deutschland repräsentative Online-Befragung von 4589 Frauen mit mindestens einem Kind unter 6 Jahren, die über eine Einwohnermeldeamtsstichprobe gewonnen wurden. In der Online-Befragung wurde nach einem Screening aller Lebenszeitschwangerschaften Fragen zu jeweils einer ausgewählten Schwangerschaft, der Fokusschwangerschaft, gestellt. Aus dieser Einwohnermeldeamtsstichprobe wählten 160 Frauen eine abgebrochene Schwangerschaft als Fokusschwangerschaft aus. Für die Befragung von Frauen mit Schwangerschaftsabbrüchen wurden weitere Zugangswege wie Abbrucheinrichtungen (253), Beratungsstellen (28), Women on Web^1^ (24), soziale Medien (186) sowie eine Sonderstichprobe (Frauen mit Gewalt- oder Fluchterfahrungen; 11) genutzt. Insgesamt beantworteten 662 Frauen mit Schwangerschaftsabbruch die Fragen der Online-Befragung, darunter befanden sich 594 Frauen mit Abbruch einer ungewollten Schwangerschaft in Deutschland, die in die folgende Auswertung eingingen. Die Stichprobe dieser 594 Frauen erfüllt nicht das Kriterium der Repräsentativität im Sinne einer Zufallsauswahl. Allerdings gleicht diese Stichprobe hinsichtlich ihrer Zusammensetzung in zentralen Merkmalen den Strukturdaten der Statistik [31] zu den Schwangerschaftsabbrüchen, die das Statistische Bundesamt aus den gemeldeten Schwangerschaftsabbrüchen erstellt.

Die Datenerhebung erfolgte von November 2021 bis September 2022. Die Daten wurden über einen zuvor durch einen Pretest validierten Fragebogen erhoben, der in 6 Sprachen (Deutsch, Englisch, Französisch, Arabisch, Farsi und Türkisch) vorlag und etwa 40–50 min für die Bearbeitung beanspruchte. Bei erfolgreichem Abschluss erhielten die Befragten eine Aufwandsentschädigung in Höhe von 20 €.

Die 5 Zugangsdimensionen nach Penchansky und Thomas wurden durch Fragen operationalisiert, wobei die Zuordnung der Fragen zu den Dimensionen nicht immer trennscharf ist. So kann es z. B. sowohl aufgrund eines schlechten Zugangs zu Informationen (Zugangsdimension Passfähigkeit) als auch aufgrund einer geringen Verfügbarkeit (Zugangsdimension Verfügbarkeit) schwierig sein, eine Abbrucheinrichtung zu finden. Anschließend wurde aus den 7 Fragen ein Index gebildet, der Zugangsbarrieren in den Dimensionen Verfügbarkeit, Erreichbarkeit, Passfähigkeit und Bezahlbarkeit einschließt.

Die statistischen Auswertungen erfolgten mithilfe des Statistikprogramms IBM SPSS Version 28 (Cramer‑V mit Chi-Quadrat-Test, 2‑seitige Tests mit 5 %-Signifikanzniveau). Signifikante Ergebnisse werden aufgrund der Stichprobenzusammensetzung als ein Indiz für die Annahme in der Grundgesamtheit gewertet.

## Ergebnisse

Im folgenden Abschnitt werden die Ergebnisse zu Barrieren in 4 Zugangsdimensionen dargestellt. Bei den Befunden zu den einzelnen Barrieren im Zugang zur Versorgung bei Schwangerschaftsabbruch wird auf die Verfügbarkeit geeigneter Angebote, deren räumliche Erreichbarkeit, auftretende Informationsbarrieren sowie organisatorische und finanzielle Herausforderungen eingegangen. Anschließend wird ein Zugangsindex vorgestellt, der die Summe der von den Frauen wahrgenommenen Barrieren auf dem Weg zur Inanspruchnahme eines Schwangerschaftsabbruchs abbildet. Dieser Index ermöglicht es, die Vielschichtigkeit der Zugangshürden in einer Kennzahl zu erfassen. Durch die Betrachtung der einzelnen Aspekte sowie des Gesamtindexes lassen sich die spezifischen Barrieren identifizieren, die Frauen den Zugang zu einem Schwangerschaftsabbruch erschweren.

### Verfügbarkeit von Einrichtungen zum Schwangerschaftsabbruch

**Abb. 1:**
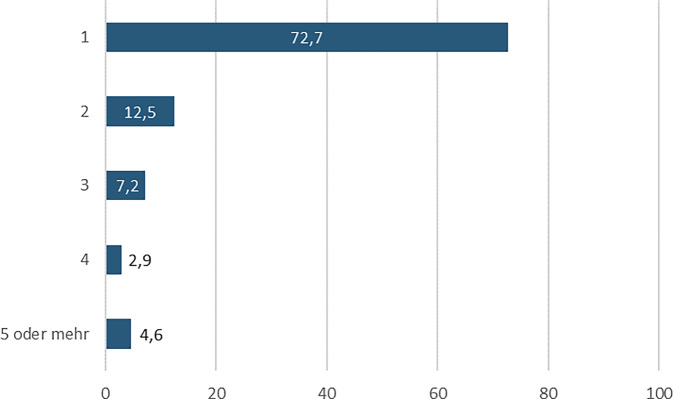
Anzahl kontaktierter Einrichtungen für einen Termin zum Schwangerschaftsabbruch (in %), *n* = 589, nur Befragte mit Abbruch einer ungewollten Schwangerschaft in Deutschland, Quelle: ELSA-Befragung von Frauen mit ausgetragenen und abgebrochenen Schwangerschaften 2022, eigene Abbildung

Für die Beschreibung der Verfügbarkeit wurden sowohl die Anzahl von Einrichtungen erfragt, die für einen Termin zum Schwangerschaftsabbruch kontaktiert werden mussten, als auch die subjektiv eingeschätzte Schwierigkeit, eine Einrichtung für einen Schwangerschaftsabbruch zu finden. Mehr als jede 4. ungewollt Schwangere musste mehr als eine Einrichtung kontaktieren, um einen Termin für einen Schwangerschaftsabbruch zu erhalten. Abb. 1 zeigt die Anzahl der Einrichtungen, die die Frauen für einen Schwangerschaftsabbruch kontaktierten. 27,3 % der Befragten gaben an, dass sie in mehr als einer Praxis wegen des Schwangerschaftsabbruchs anfragten. Von diesen musste über die Hälfte mehr als 2 Praxen kontaktieren und von diesen wiederum etwa die Hälfte sogar 4 Praxen oder mehr.

**Abb. 2:**
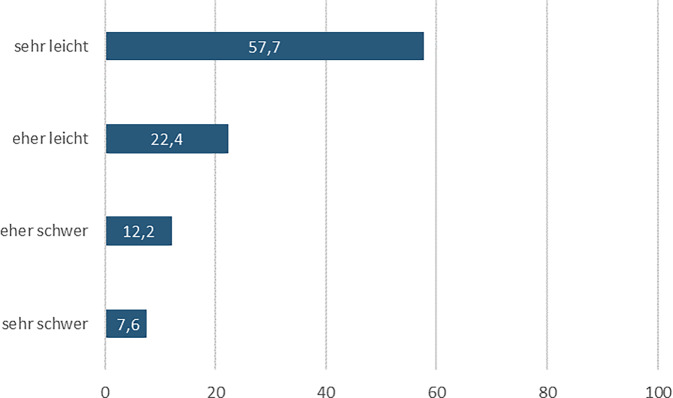
Schwierigkeit, eine Einrichtung für den Schwangerschaftsabbruch zu finden (in %), *n* = 589, nur Befragte mit Abbruch einer ungewollten Schwangerschaft in Deutschland, Quelle: ELSA-Befragung von Frauen mit ausgetragenen und abgebrochenen Schwangerschaften 2022, eigene Abbildung

Abb. 2 zeigt, dass mit 80,1 % eine deutliche Mehrheit der Teilnehmerinnen keine oder keine größeren Schwierigkeiten wahrnahm, eine geeignete Einrichtung für einen Schwangerschaftsabbruch zu finden. 57,7 % der Befragten empfanden es als sehr leicht, weitere 22,4 % als eher leicht. Allerdings sah sich auch eine nicht zu vernachlässigende Minderheit mit Herausforderungen konfrontiert. Knapp ein Fünftel (19,8 %) der Befragten stufte es als schwer oder eher schwer ein, eine Einrichtung zu finden, die einen Schwangerschaftsabbruch vornimmt.

### Räumliche Erreichbarkeit von Einrichtungen zum Schwangerschaftsabbruch

Um die Frage nach den Entfernungen zu den jeweiligen Einrichtungen für einen Schwangerschaftsabbruch zu beantworten, sollten die Teilnehmerinnen die ungefähre Distanz anhand vorgegebener Kategorien angeben. Die Befragten konnten zwischen folgenden Entfernungsbereichen wählen: bis zu 10 km, mehr als 10 km bis 50 km, mehr als 50 km bis 100 km sowie über 100 km. Ab einer Entfernung von mehr als 50 km zur Einrichtung wird der Weg als eine Zugangsbarriere bewertet. Die Auswertung ergab, dass etwas mehr als die Hälfte der Befragten (52,8 %) eine weniger als 10 km entfernte Einrichtung aufsuchte. Rund ein Drittel (32,3 %) gab eine Entfernung zwischen 10 km und 50 km an. Für 10,5 % der Teilnehmerinnen lag die Praxis oder Klinik 50 km bis 100 km von ihrem Wohnort entfernt und 4,5 % mussten eine Strecke von über 100 km zurücklegen, um einen Schwangerschaftsabbruch durchführen zu lassen.

Neben der Entfernungsangabe wurden die Teilnehmerinnen auch nach ihrer subjektiven Einschätzung zur räumlichen Erreichbarkeit der jeweiligen Abbrucheinrichtung befragt. Diese Bewertung berücksichtigt neben der Distanz weitere Faktoren wie die Anbindung an den öffentlichen Nahverkehr, die Verfügbarkeit eines Pkw oder die Möglichkeit, von Dritten gefahren zu werden. Die Mehrheit der Befragten (64,8 %) stufte die Erreichbarkeit der aufgesuchten Einrichtung als sehr gut ein. Weitere 26,2 % bewerteten sie als gut. Allerdings beurteilte ein nicht unerheblicher Anteil von 9,0 % die Erreichbarkeit als eher oder sehr schlecht. Für diese Gruppe stellte die Anreise zur Abbrucheinrichtung offenbar eine Herausforderung dar, sei es aufgrund großer Entfernungen oder mangelnder Mobilitätsmöglichkeiten. Die Gegenüberstellung der Entfernungsdaten und der subjektiven Bewertungen verdeutlicht, dass die räumliche Erreichbarkeit ein multidimensionales Konzept ist, das nicht allein von der Distanz abhängt. Faktoren wie der Zugang zu Transportmitteln und der Wohnort spielen eine ebenso große Rolle für die tatsächliche Zugänglichkeit der Versorgungsangebote.

### Informationsbarrieren

**Abb. 3:**
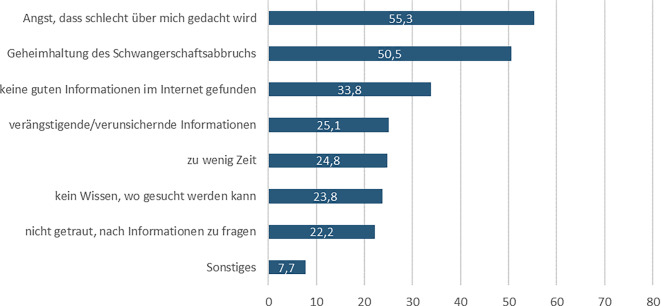
Barrieren beim Zugang zu Informationen (in %), *n* = 311, Mehrfachnennung möglich, nur Befragte mit Informationsbarrieren und Abbruch einer ungewollten Schwangerschaft in Deutschland Quelle: ELSA-Befragung von Frauen mit ausgetragenen und abgebrochenen Schwangerschaften 2022, eigene Abbildung

Die Teilnehmenden wurden nach Barrieren gefragt, die den Zugang zu Informationen beeinträchtigen können. Etwas mehr als jede 2. Befragte (57,7 %) stieß auf Informationsbarrieren. 55,3 % von ihnen gaben an, dass sie Angst hätten, dass schlecht über sie gedacht wird, und fast die Hälfte (50,5 %) aufgrund der Geheimhaltung des Schwangerschaftsabbruches (Abb. 3). Ein Drittel (33,8 %) fand es schwierig, im Internet gute Informationen zu finden. Weitere Barrieren aufgrund von verängstigenden Informationen, Zeitdruck sowie Unkenntnis über Informationszugänge und sich nicht getraut zu haben, nach Informationen zu fragen, wurden von etwa 22–25 % der Befragten angegeben.

Die Analyse der Daten ergab keinen statistisch signifikanten Zusammenhang zwischen dem Alter der Befragten und dem Auftreten von Informationsschwierigkeiten (Cramer-V = 0,111, *p* = 0,151). Daraus lässt sich schließen, dass ungewollt Schwangere unabhängig von ihrem Lebensalter mit ähnlichen Herausforderungen bei der Informationsbeschaffung konfrontiert sind. Hinsichtlich der Bildung zeigt sich ein interessanter Zusammenhang: Je höher die Bildung der Teilnehmerinnen war, desto häufiger berichteten sie von Informationsbarrieren. Dieser Effekt erwies sich als schwach, aber statistisch signifikant (Cramer-V = 0,127, *p* < 0,050).

### Organisatorische Herausforderungen im Kontext eines Schwangerschaftsabbruches

**Abb. 4:**
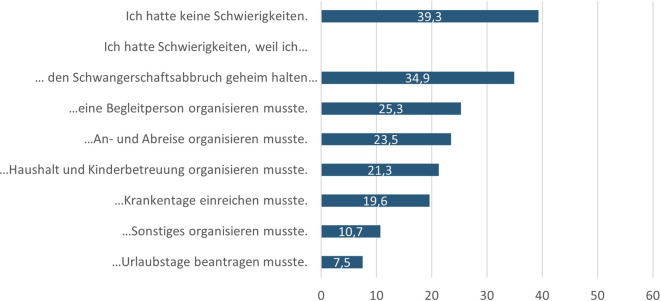
Schwierigkeiten, den Schwangerschaftsabbruch zu organisieren (in %), *n* = 588, Mehrfachnennungen möglich, nur Befragte mit Abbruch einer ungewollten Schwangerschaft in Deutschland, Quelle: ELSA-Befragung von Frauen mit ausgetragenen und abgebrochenen Schwangerschaften 2022, eigene Abbildung

Während 39,3 % der befragten Frauen keine Hürden in der Organisation des Schwangerschaftsabbruchs angeben, sah sich mit 60,7 % die Mehrheit der befragten Frauen mit diversen organisatorischen Schwierigkeiten konfrontiert (Abb. 4). Mehr als ein Drittel (34,9 %) berichtete davon, Probleme gehabt zu haben, weil der Schwangerschaftsabbruch geheim gehalten wurde. Ein Viertel (25,3 %) hatte Schwierigkeiten, eine Begleitperson für den Eingriff zu organisieren, und für 23,5 % gestalteten sich die An- und Abreise zur Einrichtung schwierig. Rund ein Fünftel (21,3 %) gab Probleme bei der Organisation von Haushalt und Kinderbetreuung an. Diese Ergebnisse verdeutlichen die Vielzahl der Aspekte, die im Zusammenhang mit einem Schwangerschaftsabbruch zu berücksichtigen sind. Soziale Faktoren wie die Geheimhaltung sowie die Vereinbarkeit mit Beruf, Familie und Haushalt spielen eine zentrale Rolle.

Während 19,0 % der Befragten nur eine Schwierigkeit angaben, berichteten 16,7 % von 2 und 14,6 % von 3 Problemen. Jede 10. Teilnehmerin (10,4 %) sah sich sogar mit 4 oder mehr Hürden konfrontiert. Insgesamt summierten sich bei 41,7 % der Befragten mehrere organisatorische Herausforderungen. Besonders häufig traten Schwierigkeiten aufgrund von Geheimhaltung, der Organisation einer Begleitperson sowie der An- und Abreise gemeinsam auf. So gaben 60,9 % der Teilnehmerinnen mit Problemen bei der An- und Abreise auch Schwierigkeiten bei der Suche einer Begleitperson an (Cramers V = 0,452, *p* < 0,001). Zudem berichteten 40,6 % dieser Gruppe von Hürden bei Haushalt und Kinderbetreuung (Cramers V = 0,262, *p* < 0,001). Weiterhin zeigte sich ein enger Zusammenhang zwischen Schwierigkeiten aufgrund der Geheimhaltung des Abbruchs und Schwierigkeiten im Kontext mit einer Krankschreibung (Cramers V = 0,314, *p* < 0,001) sowie der Suche nach Begleitpersonen (Cramers V = 0,337, *p* < 0,001). Diese Korrelationen erscheinen plausibel, da die Geheimhaltung die Suche nach einer vertrauenswürdigen Begleitperson sowie die Erklärung einer Abwesenheit erschwert.

### Bezahlbarkeit

Die Finanzierung des Schwangerschaftsabbruchs stellt für viele ungewollt Schwangere eine bedeutende Hürde dar. Die Studie untersuchte daher, wie die Teilnehmerinnen die Aufbringung der damit verbundenen Kosten subjektiv bewerten. Obwohl knapp die Hälfte (45,6 %) der Befragten es als leicht oder sehr leicht empfand, die Kosten zu tragen, und weitere 32,7 % die finanzielle Bewältigung zumindest als eher leicht einschätzten (insgesamt 78,3 % der Befragten), stellte die Finanzierung des Schwangerschaftsabbruchs für 21,9 %, und damit für jede 5. Frau, eine erhebliche Herausforderung dar.^2^

Die Kosten für den Schwangerschaftsabbruch werden für gering verdienende Frauen von den Bundesländern übernommen. Von den Befragten hatten 62,2 % eine Kostenübernahme beantragt und die Kosten für den Abbruch wurden übernommen, bei 6,7 % (*n* = 39) wurden sie abgelehnt. Fast ein Drittel (31,2 %) beantragte keine Kostenübernahme. Die Bewertung als finanzielle Belastung hängt mit der Kostenübernahme zusammen (Cramers V = 0,137; *p* < 0,001): Von den Frauen, deren Antrag auf Kostenübernahme abgelehnt wurde, gaben 46,2 % an, dass es ihnen eher oder sehr schwerfiel, die Kosten aufzubringen. Auch ein Viertel (25,2 %) derjenigen, die keine Kostenübernahme beantragt hatte, bewertete die finanzielle Belastung als eher oder sehr schwer. Im Gegensatz dazu empfanden nur 15,7 % der Frauen mit Kostenübernahme die Aufbringung der Kosten als eher oder sehr schwierig.

### Barrieren zum Schwangerschaftsabbruch – Zugangsindex

**Abb. 5:**
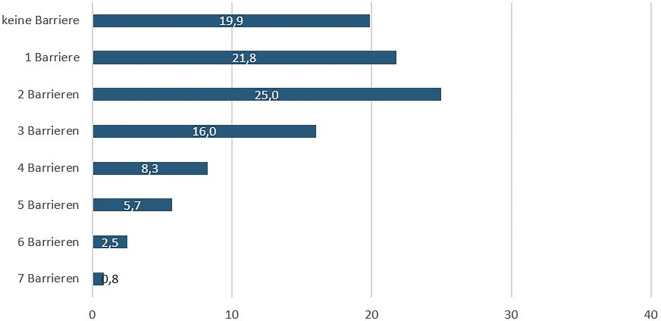
Anzahl der Barrieren beim Zugang zum Schwangerschaftsabbruch (in %), *n* = 593, nur Befragte mit Abbruch einer ungewollten Schwangerschaft in Deutschland, Quelle: ELSA-Befragung von Frauen mit ungewollten ausgetragenen und abgebrochenen Schwangerschaften 2022, eigene Abbildung

Zur Darstellung der Barrieren beim Zugang zur medizinischen Versorgung wurde aus den 7 eingeschlossenen Variablen ein einfacher Zugangsindex gebildet. Dieser Index spiegelt wider, mit wie vielen Barrieren die Frauen beim Versuch der Inanspruchnahme von Versorgungsleistungen zum Schwangerschaftsabbruch konfrontiert waren. Die Auswertung des Zugangsindexes in Abb. 5 zeigte, dass 81,1 % der Frauen von mindestens einer Hürde betroffen waren. Für mehr als die Hälfte der Frauen (58,3 %) ergaben sich mindestens 2 und für 33,3 % mindestens 3 Hindernisse auf ihrem Weg zur medizinischen Versorgung. 17,3 % der Frauen waren mit 4 oder mehr Barrieren konfrontiert.

Die Analyse der erhobenen Daten ergab keine Hinweise darauf, dass die wahrgenommenen Zugangsbarrieren mit spezifischen Merkmalen der befragten Frauen in Zusammenhang stehen. Weder das Alter noch der Bildungsgrad, der Bezug staatlicher Unterstützungsleistungen oder eine Migrationsgeschichte korrelierten in statistisch signifikanter Weise mit der Anzahl der berichteten Hürden beim Zugang zur medizinischen Versorgung.

## Diskussion

Die vorliegende Studie liefert Erkenntnisse über den Zugang zu einem Schwangerschaftsabbruch in Deutschland. Die Ergebnisse der Befragung von Frauen mit ungewollten Schwangerschaften im Rahmen der ELSA-Studie bestätigen die internationalen Erfahrungen, dass ungewollt Schwangere verschiedenen Barrieren gegenüberstehen [1, 22]. Vergleiche mit internationalen Studien, etwa von Foster [17] und Jerman et al. [2], zeigen, dass die Zugangsbarrieren in Deutschland denen ähneln, die auch in anderen Ländern beobachtet wurden [17, 28]. Die Studienergebnisse verdeutlichen, dass in Deutschland der Zugang zu einem Schwangerschaftsabbruch für viele Frauen mit Hürden unterschiedlicher Art verbunden ist. Etwa 40 % der Befragten berichteten über Barrieren in der Verfügbarkeit und Erreichbarkeit von Einrichtungen für einen Schwangerschaftsabbruch. Jede 4. Befragte berichtete von mehr als einer Barriere im Zugang, jede 10. Befragte von mehr als 2 Barrieren. Zwar bewertete mit 80,1 % eine Mehrheit der Befragten es als eher und sehr leicht, eine Einrichtung zu finden, aber knapp ein Fünftel (19,8 %) bewertete die Suche als eher oder sehr schwer. Mehr als jede 4. Befragte musste mehr als eine Einrichtung kontaktieren, um einen Termin für einen Schwangerschaftsabbruch zu bekommen. Abgeleitet aus den Ergebnissen können verschiedene Gründe dazu führen, dass ungewollt Schwangere mehrere medizinische Einrichtungen aufsuchen müssen, bevor ein Schwangerschaftsabbruch durchgeführt werden kann. Einerseits kann ein Informationsdefizit bezüglich der verfügbaren Abbrucheinrichtungen vorliegen, sodass zunächst Einrichtungen kontaktiert werden, die keine Schwangerschaftsabbrüche anbieten. Infolgedessen müssen nach einer Absage weitere Einrichtungen angefragt werden. Andererseits kann es vorkommen, dass die kontaktierten Einrichtungen nicht über ausreichende Kapazitäten verfügen, um den Eingriff innerhalb der rechtlich vorgegebenen Frist oder mit der gewünschten Methode durchzuführen. In solchen Fällen sind ebenfalls Mehrfachanfragen erforderlich, bis eine geeignete Einrichtung gefunden wird, die den Schwangerschaftsabbruch termingerecht und nach den Präferenzen der Schwangeren vornehmen kann. Diese Ergebnisse zeigen, dass hier trotz der mehrheitlich positiven Einschätzung Verbesserungspotenzial hinsichtlich der Zugänglichkeit von Abbrucheinrichtungen besteht.

Die räumliche Erreichbarkeit von Einrichtungen für Schwangerschaftsabbrüche wurde sowohl über objektive Entfernungsdaten als auch über die subjektive Bewertung der Teilnehmerinnen erhoben. Ein kleinerer Anteil von 9,0 % beurteilte die Erreichbarkeit als eher oder sehr schlecht. Für diese Gruppe stellte die Anreise zur Abbrucheinrichtung eine Hürde dar, sei es aufgrund weiter Entfernungen, unzureichender Verkehrsanbindung oder mangelnder Mobilitätsmöglichkeiten. Hinsichtlich der objektiven Entfernungsangaben gaben 15,0 % der Teilnehmerinnen an, dass die Einrichtung für den Schwangerschaftsabbruch über 50 km entfernt war. Die Gegenüberstellung der Entfernung mit den subjektiven Bewertungen verdeutlicht, dass die räumliche Erreichbarkeit ein multidimensionales Konzept ist, das nicht allein von der Distanz abhängt. Faktoren wie die Verfügbarkeit von Transportmitteln spielen eine ebenso große Rolle für die tatsächliche Zugänglichkeit der Versorgungsangebote. Eine gute Verfügbarkeit und Erreichbarkeit sind Voraussetzungen für eine niedrigschwellige Abbruchversorgung [1].

Die Studie untersuchte Probleme, mit denen ungewollt Schwangere bei der Suche nach Informationen zum Schwangerschaftsabbruch konfrontiert waren, und kommt zu dem Ergebnis, dass 58,1 % der Teilnehmerinnen auf Schwierigkeiten bei der Informationssuche stießen. Die häufigsten Hindernisse waren Angst vor negativer Beurteilung (54,4 %), Informationsdefizite aufgrund von Geheimhaltung (49,7 %) und Schwierigkeiten, qualitativ hochwertige Informationen im Internet zu finden (33,2 %). Interessanterweise berichteten Frauen mit höherem Bildungsstand häufiger von Informationsbarrieren, möglicherweise aufgrund eines kritischeren Blicks auf die Informationsqualität und höherer Ansprüche. Insgesamt zeigen sich viele Barrieren beim Zugang zu Informationen. Diese Barrieren können längere Wartezeiten, höhere Kosten, größeren organisatorischen Aufwand sowie Stigmatisierungserfahrungen aufgrund erzwungener Aufdeckung zur Folge haben [2, 3, 16, 27, 28]. Insgesamt unterstreichen diese Ergebnisse die Notwendigkeit, unabhängig von Alter und Bildungshintergrund barrierefreien Zugang zu hochwertigen Informationen rund um das Thema Schwangerschaftsabbruch zu gewährleisten, was auch den Wünschen von Frauen entspricht [7, 29]. Nur so können ungewollt Schwangere eine fundierte und selbstbestimmte Entscheidung treffen [1].

Der Großteil der Befragten (60,7 %) sah sich auf dem Weg zu einem Schwangerschaftsabbruch mit diversen organisatorischen Schwierigkeiten konfrontiert. Im Durchschnitt wurden 3 verschiedene Probleme angegeben. Lediglich 39,3 % gaben an, keinerlei Hürden dieser Art erlebt zu haben. Etwa ein Drittel der Befragten (34,9 %) gab an, dass der Wunsch oder die Notwendigkeit, die Schwangerschaft oder den geplanten Abbruch geheim zu halten, zu organisatorischen Schwierigkeiten führte. Neben der Frage zu Versorgungsangeboten spielen auch soziale Faktoren wie Stigmatisierung, Geheimhaltung sowie die Vereinbarkeit mit Beruf, Familie und Haushalt eine zentrale Rolle.

Die Kosten stellen für viele ungewollt Schwangere eine bedeutende Hürde bei der Inanspruchnahme eines Schwangerschaftsabbruchs dar. 21,9 % der befragten Frauen bewerteten es als eher oder sehr schwer, die erforderlichen Mittel aufzubringen. Dies trifft insbesondere Frauen, deren Antrag auf Kostenübernahme abgelehnt wurde (46,2 %), aber auch Frauen, die keine Kostenübernahme beantragt hatten (25,2 %). Diese Ergebnisse verdeutlichen die ökonomische Dimension, die ein Schwangerschaftsabbruch für viele Betroffene hat. Neben den rein medizinischen Kosten können zusätzliche Ausgaben beispielsweise für Anreise, Unterbringung oder Ausfallzeiten am Arbeitsplatz anfallen.

Die Summe der Zugangsbarrieren zeigt, dass mit 19,2 % ein geringer Teil der Befragten keine Barriere beim Zugang zu einem Schwangerschaftsabbruch erlebte. Die Gründe für Hindernisse bei der Inanspruchnahme von Gesundheitsleistungen sind vielschichtig und lassen sich nicht auf einzelne soziodemografische Faktoren reduzieren. Möglicherweise spielen stattdessen strukturelle Aspekte des Gesundheitssystems sowie individuelle Lebensumstände eine größere Rolle. Denkbar sind beispielsweise eine unzureichende regionale Versorgungsdichte, mangelnde Informationen über Angebote oder bürokratische Hürden, die den Zugang erschweren.

### Stärken und Limitationen

Im Rahmen der ELSA-Studie ermöglichten vor allem die Praxispartner*innen den Zugang zu Frauen mit Schwangerschaftsabbruch. Dies gelang trotz der durch die COVID-19-Pandemie bedingten Zugangsbeschränkungen im Feld, allerdings variierte die Gewinnung von Teilnehmenden aus Abbrucheinrichtungen und Beratungsstellen erheblich, abhängig von der Initiative des dortigen Personals. Des Weiteren erfordert die Nutzung von Social-Media-Kampagnen zur Teilnehmergewinnung eine kritische Betrachtung möglicher Selektionseffekte, insbesondere hinsichtlich der Erreichbarkeit von Frauen mit begrenzten Deutschkenntnissen oder niedrigerer Bildung. Um diesen Herausforderungen zu begegnen, wurde besonderes Augenmerk auf die Gestaltung klar verständlicher Anschreiben und Fragebögen sowie auf die Bereitstellung mehrsprachiger Dokumente gelegt, um die Inklusion dieser Zielgruppe zu verbessern. Ein Bias lässt sich durch sozial erwünschtes Antwortverhalten dennoch nicht vollständig ausschließen, wenngleich erwartet wird, dass die Anonymität der Befragungen und die sorgfältige Formulierung der Fragen zu einer Reduzierung dieses Effekts führen sollten. Ungewollt Schwangere mit Gewalterfahrungen in der Partnerschaft wurden in dieser Auswertung nicht betrachtet, weil sie Gegenstand eines Teilprojektes sind.

Der standardisierte Online-Fragebogen mit vorgegebenen Antwortoptionen ermöglicht eine hohe Objektivität und Reliabilität bei der Datenerhebung und -auswertung [30]. Die gesamtgesellschaftliche Tabuisierung des Schwangerschaftsabbruchs könnte Frauen allerdings davon abgehalten haben, selbst im Rahmen einer anonymen Studie Schwangerschaftsabbrüche anzugeben oder Fragen hierzu zu beantworten. Letztlich ist durch die Online-Befragung nicht auszuschließen, dass auch andere Personen, die selbst nicht zur Zielgruppe gehörten, an der Befragung teilnahmen. Durch die sehr spezifischen Zugangswege und Fragestellungen sowie die Fragebogenlänge wird dieses Risiko jedoch als gering eingeschätzt. Die Stichprobe wurde darüber hinaus mit den Daten zur Statistik über Schwangerschaftsabbrüche des Statistischen Bundesamts verglichen, welche eine gute Vergleichsquelle für die Einschätzung der Datengüte darstellen. Die gewonnenen Daten spiegeln insgesamt gut die Strukturdaten der Meldestatistik für Schwangerschaftsabbrüche des Statistischen Bundesamtes wider. Die zuvor durchgeführten Pretests lieferten außerdem gute Hinweise darauf, die Validität der Daten sicherzustellen.

## Fazit

Die Studienergebnisse unterstreichen die Notwendigkeit in Deutschland, den Zugang zu sicheren Schwangerschaftsabbrüchen gemäß der WHO-Leitlinie zu verbessern [1]. Viele Teilnehmerinnen sahen sich mit verschiedenen Barrieren beim Zugang zum Schwangerschaftsabbruch konfrontiert. Ungewollt Schwangere erleben im Zusammenhang mit einem Schwangerschaftsabbruch nicht selten Zugangsbarrieren, die vermeidbar sind. Neben der reinen Verfügbarkeit von Abbrucheinrichtungen zeigten sich Hürden bei der Informationsbeschaffung, der räumlichen Erreichbarkeit, der Finanzierung sowie organisatorischen Aspekten. Besonders die Geheimhaltung des Eingriffs und damit einhergehende Stigmatisierungsängste stellten für viele Teilnehmerinnen Hürden dar. Die WHO betont das Recht von Frauen auf einen zeitnahen Zugang zu qualitativ hochwertigen und bezahlbaren Abbruchleistungen ohne Diskriminierung [1]. Die vorliegenden Daten deuten jedoch darauf hin, dass dieses Recht in Deutschland nach wie vor nicht uneingeschränkt gewährleistet ist. Viele der genannten Barrieren stehen im Zusammenhang mit den geltenden rechtlichen Regelungen. Zum Abbau der Zugangsbarrieren gehören daher die Entkriminalisierung des Schwangerschaftsabbruches, eine flächendeckende Versorgungsstruktur, finanzielle Unterstützungsangebote, verbesserte Informationsangebote sowie der Abbau von Stigmatisierung und Diskriminierung [1, 6]. Die WHO-Leitlinie empfiehlt, den Schwangerschaftsabbruch als Gesundheitsleistung einzuordnen, dessen Kosten die Krankenkassen übernehmen, um einen niedrigschwelligen Zugang zu sichern [1]. Nur wenn alle Dimensionen der Zugänglichkeit berücksichtigt und Barrieren abgebaut werden, kann das Recht auf einen sicheren Schwangerschaftsabbruch unabhängig von sozioökonomischem Status oder Lebenssituation gewährleistet werden.

## Footnotes

1 Women on Web ist eine kanadische gemeinnützige Organisation, die Informationen und Unterstützung für Frauen anbietet, die einen Schwangerschaftsabbruch benötigen. Sie versendet Medikamente zum Schwangerschaftsabbruch per Post und ermöglicht dadurch telemedizinische Abbrüche in mehr als 200 Ländern, insbesondere in Ländern, wo diese rechtlich eingeschränkt oder gefährlich sind.

2 Kosten des Abbruchs und weitere Kosten: Fast jede dritte der befragten Frauen beantragte keine Kostenübernahme, sondern zahlte die Kosten des Schwangerschaftsabbruchs selbst. Selbstzahlerinnen, dazu zählen auch Frauen, deren Kostenübernahme abgelehnt wurde, bezahlen unterschiedliche Beträge für einen Schwangerschaftsabbruch: 9,1 % der Selbstzahlerinnen zahlten bis zu 200 €, 53,1 % zwischen 201 € und 399 €. 30,1 % der Frauen gab Kosten in Höhe von 400–599 € an und 7,7 % zahlten 600 € und mehr. 54,6 % der Befragten gaben an, dass keine zusätzlichen Kosten über den Abbruch hinaus angefallen sind (zum Beispiel Kosten für An- und Abreise, Verdienstausfall, Kinderbetreuung).
